# Supplementary material for: MetaRibo-Seq measures translation in microbiomes
Source: Nat Commun. 2020 Jun 29;11:3268. doi: 10.1038/s41467-020-17081-z (PMC7324362; doi:10.1038/s41467-020-17081-z)
Supplement: Supplementary file 10 — Supplementary Data 7 [file 41467_2020_17081_MOESM10_ESM.zip › File2/Confidence_VeryHigh_Taxonomy/39618_out.krona.html]

Javascript must be enabled to view this page.

members
magnitude
magnitudeUnassigned
count
unassigned
taxon
rank

39618\_out

4

superkingdom
4
2

1239
4
phylum

class
4
186801

1
4
186802
order

SRS148511\_contig\_number\_contig-100\_12154.12155

2
family
186803

genus
2
1506553

1
species

SRS146888\_contig\_number\_10231
1531


SRS143598\_contig\_number\_2980
208479
1
species

family
1
31979

1485
genus
1

1
species

SRS021153\_contig\_number\_contig-100\_12309.12309
1263065
